# Infant Feeding

**Published:** 1909-09

**Authors:** 


**Infant Feeding.** By J. S. FOWLER, M.D. Pp. x, 230. London: Oxford Medical Publications. 1909.

**Diet in Infancy.** By A. DINGWALL FORDYCE, M.D. Pp. x, 174. Edinburgh: William Green & Sons. 1908.

Side by side with a diminishing birth-rate we see an immense increase in the number of books on infant feeding and the diseases of children, till one may imagine that eventually the output of the latter may equal or exceed that of the former. Meanwhile, we may observe that in general the teaching inculcated in the numerous volumes on infant feeding, though excellent, is apt to be monotonous. It is, however, a consolation to reflect that even on the basis of a very moderate sale for each individual volume, every qualified man will soon, in all probability, be in possession of a manual giving explicit directions on the subject of the rearing of infants, and insisting on the importance of the general principles on which all such directions are based.

Dr. Fowler's book is in many respects an excellent example of its kind. The style is clear and explicit, the directions very practical, and the element of personal experience well marked throughout. We have read it with pleasure, notwithstanding the fact that our appetite for these works has become a trifle jaded of late. Dr. Fowler advocates sterilisation or boiling of milk as being the safest course under the present admittedly unsatisfactory conditions of milk supply. He somewhat discounts the disadvantages of this procedure, and is inclined to regard the fear of scorbutus as exaggerated. We should have preferred a stronger plea for the purity of the milk supply, and are not inclined to minimise the danger of scorbutic and allied conditions. He is partly converted to the late Professor Budin's whole-milk method, but gives certain very excellent and practical directions as to detail, without which no attempt should be made to apply it. We feel, however, that its general adoption would lead to a considerable increase in the amount of stale and dirty milk supplied to the consumer, and to a general set back in the matter of clean milk production. Generally, too, we think the dilutions recommended for healthy infants a little too low. We are pleased to note that, though approving of a percentage method in general, Dr. Fowler entirely condemns on practical and theoretical grounds the absurd lengths to which the method has been taken in certain quarters. As a minor point we think it a pity that the scheme of protein nomenclature recommended by the committee of the Physiological Society has not been adopted. On the whole, however, we can thoroughly recommend this book, which is not too elaborate for ordinary readers, and sufficiently detailed, on the other hand, to be practically useful. The printing and paper are excellent, and the price (5s.) very moderate.

Of Dr. Dingwall-Fordyce's little book we may say that we have read the appendices with interest. They occupy nearly half the book, and are reprints or reproductions of matter which has already appeared elsewhere. They deal with the subjects of prolonged lactation, the relation of diet to thyroid activity, laboratory results from the artificial digestion of varieties of milk, the examination of infants' stools and stomach contents, and many details as to the production and distribution of milk in Great Britain and elsewhere. The author in the main body of the book has adopted a rather curious plan of numbering his paragraphs, and in the first part of supplying italicised titles to each. We cannot say that we find the effect pleasing. It gives the impression of a series of lecture notes, though the style is not sufficiently condensed to support that supposition. We note that Budin's method is summarily condemned, and though advocating rather higher dilutions (we think quite rightly) the author adopts a very similar attitude towards the question of percentage feeding as does Dr. Fowler.

